# Prokaryotic aminopeptidase activity along a continuous salinity gradient in a hypersaline coastal lagoon (the Coorong, South Australia)

**DOI:** 10.1186/1746-1448-6-5

**Published:** 2010-04-30

**Authors:** Thomas Pollet, Mathilde Schapira, Marie-Jeanne Buscot, Sophie C Leterme, James G Mitchell, Laurent Seuront

**Affiliations:** 1 School of Biological Sciences, Flinders University, GPO Box 2100, Adelaide SA 5001, Australia; 2 UMR CARRTEL, Centre Alpin de Recherche sur les Réseaux Trophiques des Ecosystèmes Limniques, Station d'Hydrobiologie Lacustre, Université de Savoie, 75 avenue de Corzent, BP 511, 74203 Thonon les Bains Cedex, France; 3 Southern Ocean Group, Dept. of Zoology & Entomology, Rhodes University, PO Box 94, Grahamstown 6140, South Africa; 4 South Australian Research and Development Institute, Aquatic Sciences, West Beach SA 5022, Australia; 5 Center for Polymer Studies, Department of Physics, Boston University, 590 Commonwealth Avenue, Boston, MA 02215, USA; 6 Centre National de la Recherche Scientifique, France

## Abstract

The distribution and aminopeptidase activity of prokaryotes were investigated along a natural continuous salinity gradient in a hypersaline coastal lagoon, the Coorong, South Australia. The abundance of prokaryotes significantly increased from brackish to hypersaline waters and different sub-populations, defined by flow cytometry, were observed along the salinity gradient. While four sub-populations were found at each station, three additional ones were observed for 8.3% and 13.4%, suggesting a potential modification in the composition of the prokaryotic communities and/or a variation of their activity level along the salinity gradient. The aminopeptidase activity highly increased along the gradient and salinity appeared as the main factor favouring this enzymatic activity. However, while the aminopeptidase activity was dominated by free enzymes for salinities ranging from 2.6% to 13.4%, cell-attached aminopeptidase activity was predominant in more saline waters (i.e. 15.4%). Changes in substrate structure and availability, strongly related to salinity, might (i) modify patterns of both aminopeptidase activities (free and cell-associated enzymes) and (ii) obligate the prokaryotic communities to modulate rapidly their aminopeptidase activity according to the nutritive conditions available along the gradient.

## Findings

Dissolved proteins and peptides are important sources of energy and nitrogen in aquatic systems [1,2], but they must be hydrolysed to amino acids and oligopeptides to be useable by prokaryotes. Following the development of sensitive methods using fluorogenic substrates [3], proteolytic activity in natural aquatic systems has been assessed by measuring the activity of leucine-aminopeptidase as a model enzyme [4]. However, microbial cells living in aquatic systems are influenced by a variety of environmental factors which affect the molecular control of their enzyme synthesis. Among these variables, salinity has been identified as a major driving force in both the composition of bacterioplankton and their efficiency in degrading dissolved organic carbon (DOC) [5]. Previous studies focusing on the effect of salinity on the composition and metabolic activity of bacterial communities were mainly conducted in estuaries where salinity typically did not exceed 5% [6] and the effect of higher salinity conditions was mainly investigated in highly saline ponds from solar salterns [7]. To our knowledge, little is still known about the dynamic of prokaryotic aminopeptidase activity along natural continuous hypersaline gradients. The objective of this study was to investigate the changes in aminopeptidase activity of prokaryotic communities identified using flow cytometry from brackish to hypersaline waters.

**Figure 1:**
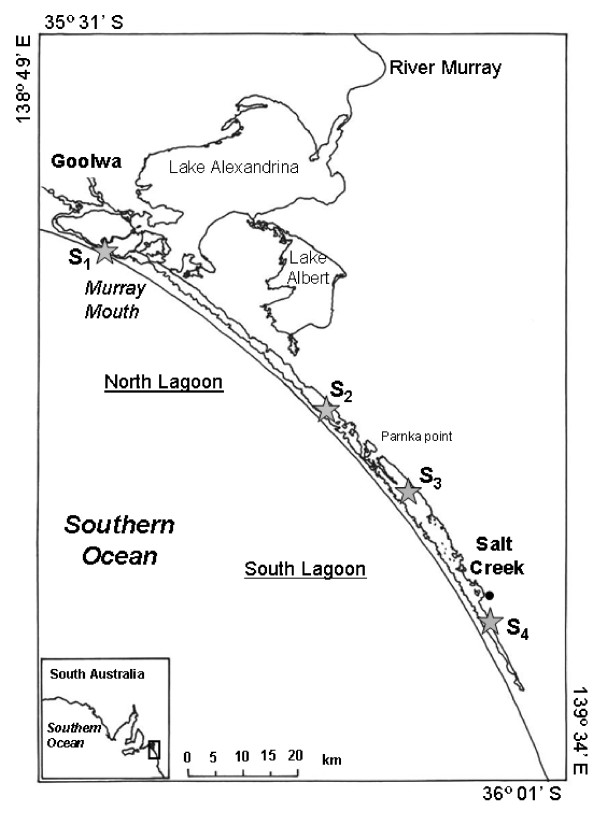
**Study site (the Coorong, South Australia) and the 4 sampling stations (✩) from S_1 _to S_4_**.

The Coorong is a South Australian shallow coastal lagoon characterized by a strong salinity gradient with salinity continuously ranging from brackish (1.8%) to hypersaline (15.5%). Constrained between the last interglacial dune and the modern dune that has been established from the mid-holocene, this lagoon receives inputs from the ocean through the Murray Mouth and from underground and freshwater inputs from Lake Alexandrina and Lake Albert, which are the terminal system of the River Murray (Fig. 1). If freshwater inputs lead to lower salinities in the northwest part of the Coorong, the excess in evaporation over precipitation increases salinity along the north-south axis, especially during the summer period.

Sub-surface samples were collected at 4 stations (S_1_, S_2_, S_3 _and S_4_; Fig. 1) characterized by increasing salinities, i.e. 2.6%, 8.3%, 13.4% and 15.4%. Temperature (°C), conductivity (mS cm^-1^) and dissolved oxygen concentrations (DO; mg l^-1^) were recorded using a YSI 85 (Fondriest) multiparameter probe. Salinity (%) was calculated from temperature and conductivity following Fofonoff and Millard [8]. Water samples were collected at each station using acid-washed 1-liter borosilicate bottles.

Water temperature ranged between 25.2 and 27.7°C. DO concentrations decreased from 3.46 mg l^-1 ^in S_1 _to 1.48 mg l^-1 ^in S_4 _(Table 1). Concentration of suspended particular matter (SPM), determined following Hewson et al [9], increased from 38 mg l^-1 ^at S_1 _to 540 mg l^-1 ^at S_4 _(Table 1). Nutrient concentrations were determined in the field using a portable LF 2400 photometer according to standard colorimetric methods for  (Indophenol blue),  (Naphtylethylene diamine),  (Naphtylethylene diamine after zinc reduction) and  (Ascorbic acid reduction). Ammonium was the most abundant form of nitrogen with concentrations consistently increasing from 2.5 μM in S_1 _to more than 110 μM in S_3 _and S_4_. Phosphate concentrations were low at S_1_, S_2 _and S_3 _(i.e. < 8.5 μM) whereas S_4 _was characterized by high phosphate concentrations (> 50 μM). Chl *a *(μg l^-1^), determined following Strickland and Parson [10] using a Turner 450 fluorometer after extraction in methanol of the samples collected on glass-fiber filters, were low at S_1_, S_2 _and S_4 _(i.e. < 3 μg l^-1^). In contrast, S_3 _was characterized by relatively high Chl *a *concentrations (13.5 μg l^-1^; Table 1).

**Table 1:** Physical and chemical parameters measured along the salinity gradient.

Parameters	S_1_	S_2_	S_3_	S_4_
S (%)	2.6	8.3	13.4	15.4
T (°C)	27.7	24.2	26.6	25.2
DO (mg l^-1^)	3.4	3.7	2.2	1.4
[NH_4_^+^] (μM)	2.5	5.3	> 110	> 110
[NO_3_^-^] + [NO_2_^-^] (μM)	< 1.6	2.9	< 1.6	1.7
[PO_4_^3-^] (μM)	1.0	8.4	1.0	> 50
[Chl *a*] (μg l^-1^)	1.2	2.5	13.5	1.3
SPM (mg l^-1^)	38.0	182.7	477.0	540.0

Salinity (S; %) and temperature (T; °C). Dissolved oxygen (DO; mg l^-1^), ammonium ([NH_4_^+^]; μM), nitrate + nitrite ([NO_3_^-^] + [NO_2_^-^]; μM), phosphate ([PO_4_^3-^]; μM), Chlorophyll *a *([Chl *a*]; μg l^-1^) and Suspended Particulate Matter (SPM; mg l^-1^) concentrations

Prokaryotic populations were identified and enumerated by flow cytometry (FCM) using a FACScanto flow cytometer. Samples were fixed and prepared following Brussaard [11]. Sub-populations were discriminated based on the differences in SYBR-I Green fluorescence and right-angle light scatter (SSC). Fluorescent beads 1 μm in diameter were added to all samples as an internal standard. Working bead concentrations were estimated after each FCM session under epifluorescent microscopy to ensure reliability of the bead concentration and all FCM parameters were normalized to bead concentration and fluorescence. Finally, populations were identified and enumerated using WinMDI 2.9 (^©^Joseph Trotter) flow cytometry analysis software. No significant differences were found between FCM counts and epifluorescence microscopy (EM) counts conducted at each station (Wilcoxon-Mann-Whitney U-test, n = 5, p > 0.05).

**Figure 2:**
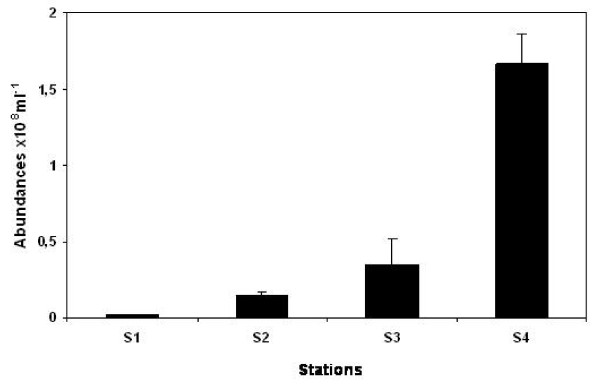
**Prokaryotic abundances (×10^8^ml^-1^) along the salinity gradient**. The error bars are the standard deviations.

**Figure 3:**
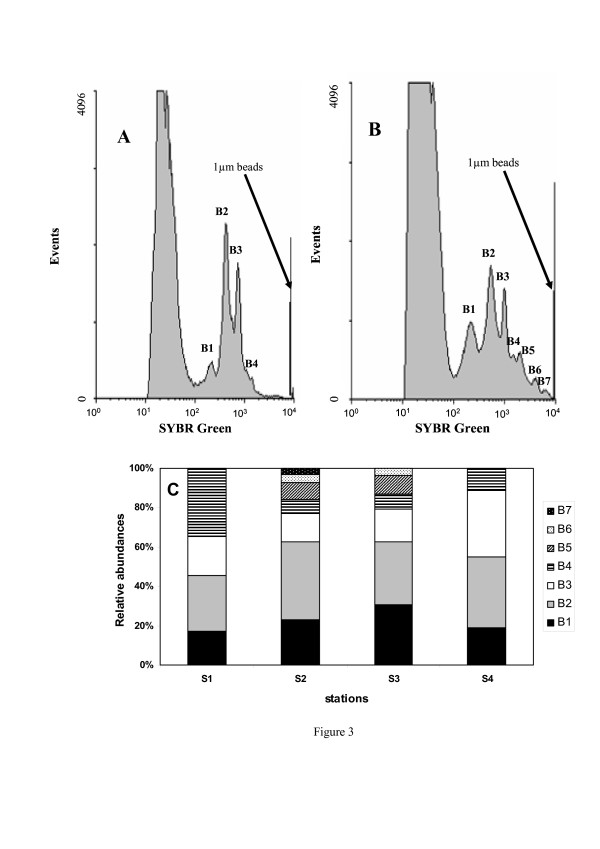
**Cytometric differentiation of prokaryotic populations**. (A) Shows results obtained from S4. Four prokaryotic sub-populations were identified; the histogram plot of green fluorescence shows 4 peaks relating to sub-populations of increasing DNA content (B1 to B4). (B) Shows results obtained from S2. Seven prokaryotic sub-populations were identified; the histogram plot of green fluorescence shows 7 peaks relating to sub-populations of increasing DNA content (B1 to B7). Sub-populations differed through their green fluorescence and side scatter, and therefore were not classified into high and low DNA-subpopulations but as different discrete populations. (C) Relative abundances (%) of cytometrically-defined sub-populations along the salinity gradient from S_1 _to S_4_.

In accordance with previous observations from solar salterns [12], the abundance of prokaryotes showed a significant increase with salinity (p < 0.05), with values ranging from 2.1 × 10^6 ^ml^-1 ^at S_1 _to 1.7 × 10^8 ^ml^-1 ^at S_4 _(Fig. 2). The high SPM and phosphate concentrations observed at the hypersaline station (S_4_) are favourable to high microbial abundance [13-17]. In addition, the decrease in viral lysis and bacterivory as well as the rapid growth of bacteria under high salinity conditions (i.e. > 15%) [12,18], might also have contributed to the high prokaryotic abundance observed in the hypersaline part of the lagoon. The prokaryotic cytometric richness also varied along the gradient (Fig. 3). Four to seven discrete sub-populations of prokaryotes were identified along the salinity gradient. Four distinct sub-populations were observed at S_1 _(S = 2.6%) and S_4 _(S = 15.4%) (Fig. 3A) whereas 7 and 6 sub-populations were identified at S_2 _(S = 8.3%) and S_3 _(S = 13.4%), respectively (Fig. 3B). The variability of the prokaryotic cytometric richness observed along the salinity gradient (Fig. 3C) could reflect a modification of both bacterial populations and their activity level [19]. It is now well known that salinity represents one of the main factors structuring the distribution of prokaryotic assemblages, favouring the dominance of some groups adapted at a particular salt concentration [20]. Along the Coorong, the highest cytometric richness was observed at 8.4% (7 sub-populations, station S_2_) and 13.4% (6 sub-populations, station S_3_). In contrast, the cytometric richness was much lower under hypersaline conditions (S_4_) and in the brackish area (S_1_). The elevated nutrient, Chl *a *and SPM concentrations recorded in this part of the lagoon, may have favoured this richness through a strong resource competition which is known to prevent the proliferation and the dominance of some communities. However, the variability in cytometric richness observed along the gradient could also reflect a change in prokaryotic activity level with salinity. This is consistent with previous results [21] showing that salinity selectively affects certain groups leading to a marked modification in their growth efficiency and cell-specific activity.

Aminopeptidase activity was estimated using the fluorogenic substrate analog, L-leucine-4-methyl-coumarinyl-7amide (Leu-AMC). AMC fluorescence was determined at 340 nm (excitation) and 440 nm (emission), with a spectrofluorometer (Hitachi Fluorescence Spectrophotometer, Model F-3000) previously calibrated. Total enzymatic activity (i.e. free enzymes dissolved in water and cell surface bound enzymes) and free extracellular enzymatic activity were estimated for each sampling site. For free enzymatic activity, water samples were previously gravity filtered through 0.2 μm pore size filters. Before each spectrophotometry analysis, subsamples without substrates were used as blanks to determine the background fluorescence of the samples at each sampling station. Aminopeptidase activity was quantified through Michaelis-Menten kinetic parameters: the highest rate of substrate hydrolysis *V*_*max *_(μM h^-1^) and the half-saturation constant for the enzyme *K*_*m *_(μM), which indicates the enzyme affinity to the substrate.

**Figure 4:**
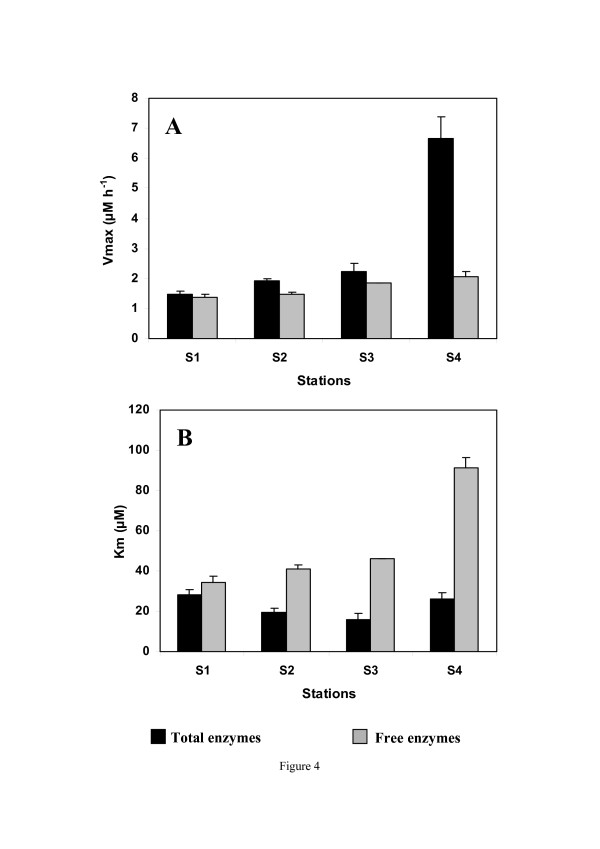
**Aminopeptidase activity along the salinity gradient**. (A) maximum enzymatic velocity, *V*_*max *_(μM h^-1^), and (B) affinity with the substrate, *K*_*m *_(μM) from S_1 _to S_4_. Total enzymatic and free enzymatic activities are shown in black and grey, respectively. The error bars represent the standard deviations.

In the present study and as previously described in solar salterns [7], the aminopeptidase activity of prokaryotes increased with salinity (Fig. 4A). The significant positive correlation observed between abundance and aminopeptidase activity is consistent with previous observations [7,22]. Specifically, the increase in the potential activity (*V*_*max*_) from station S_1 _to station S_4 _indicates that hydrolysis rates increase with salinity. In addition, given the increase in SPM concentrations by more than one order of magnitude between stations S_1 _and S_4 _(Table 1) and to the extent that prokaryotic metabolism reflects the ambient substrate availability, this increase in aminopeptidase activity suggests that the quality of organic matter may strongly differ along the salinity gradient and may indicate the existence of a gradient in the protein availability from brackish to hypersaline stations. This hypothesis is congruent with previous observations [23,24] showing that dissolved organic nitrogen (DON) and protein concentrations increased southwards along the Coorong. While this issue is well beyond the objectives of the previous work, information on the quality and quantity of organic matter would be a step forward in the understanding of the role played by salinity in prokaryotic metabolic activity. The observed increase in aminopeptidase activity beyond 15% (Fig. 4) contradicts previous results suggesting that salinity greater than 12% hampers aminopeptidase activity [7]. This difference may be related to the uniqueness of the Coorong which is characterized by a strong continuous salinity gradient with specific dynamics and functional performance of bacterioplankton communities in contrast to solar salterns considered as steady-state ecosystems with well-adapted and established communities. Further work would nevertheless be needed to confirm and generalize this potential fundamental difference between solar salterns and hypersaline lagoons.

Free and cell-associated aminopeptidase activities exhibit different patterns in relation to salinity. From 2.6% to 13.4%, free aminopeptidase activity seems to be favoured; for higher salinity (15.4%), cell-associated aminopeptidase activity is preferred (Fig. 4A). Under the assumption that protein substrates were likely more available at the hypersaline station (S_4_), this observation is congruent with Hollibaugh and Azam 's conclusions[25]. Free enzymes could be less important in the protein degradation and close physical association between prokaryotes and proteins would be necessary for efficient protein degradation. This may explain the dominance of cell-associated aminopeptidase activity observed at the hypersaline station. In addition, the changes in ionic strength related to salinity might affect the structure of substrate molecules and consequently the activity of extracellular enzymes [5]. Indeed, the solubility of proteins is known to be profoundly affected by the ionic strength and particularly by the presence of divalent cations [25]. This low solubility of proteins under hypersaline conditions might favour the prokaryote/protein association and might thus explained the higher cell-associated aminopeptidase activity observed at the hypersaline station. Moreover, the increase in SPM concentration along the salinity gradient may also favour the creation of microscale environments leading to local hotspots of prokaryotes attached to particles [26]. This may also explained the observed transition from free to cell-attached aminopeptidase activity along the gradient (Fig. 4A) and the increase in substrate affinity observed at station S_4 _(Fig. 4B) for high salinity values. It is finally stressed that free-aminopeptidase activity is unlikely to have been contaminated by cell lysis, hence over-estimated, because of the non-destructive gravity filtration conducted here. Note, however, that at the highest salinity, free enzymes might aggregate with particulate matter, leading to an overestimation of the cell-associated enzymatic activity. While this is beyond the aims of the present work, further work is needed to assess the contribution of salinity in bounding free enzymes to particulate material.

In accordance with previous reports, this first study performed along a continuous salinity gradient has shown that the increase in salinity appeared as the main factor favouring aminopeptidase activity. However, both aminopeptidase activities (free and cell-associated enzymes) are also influenced by the availability and structure of suspended materials that is susceptible of strong changes along the salinity gradient. Prokaryotic communities have then to rapidly modulate their aminopeptidase activities to optimize their fitness in response to the variability of the nutritive conditions along the salinity gradient.

Given the key role played by microbial communities in the functioning of aquatic systems, these results stress the need to extend our knowledge concerning the effect of salinity on the dynamics and activity of microbial communities in natural systems particularly in the context of global change which particularly affects local ecosystems, such as the Coorong, through changes in salinity related to modifications of freshwater discharge and evaporation. Further work is thus needed to assess the interplay between salinity and the global enzymatic activity of prokaryotic communities.

## Competing interests

The authors declare that they have no competing interests.

## Authors' contributions

TP conducted the sampling experiments, performed the abundance and aminopeptidase activity analyses and drafted the manuscript. MS conducted the sampling experiments, participated in abundance and enzymatic activity analyses and drafted the manuscript. MJB and SCL participated in sampling experiments. JGM participated in the design of study. LS designed the study, conducted the sampling experiments, and drafted the manuscript. All the authors have read and approved the final manuscript.
